# Developing a diagnostic framework for patients presenting with Exercise Induced Leg Pain (EILP): a scoping review

**DOI:** 10.1186/s13047-023-00680-6

**Published:** 2023-11-21

**Authors:** Fatma Bosnina, Nat Padhiar, Stuart Miller, Krishna Girotra, Chrysovalanto Massoura, Dylan Morrissey

**Affiliations:** 1 Sports & Exercise Medicine, William Harvey Research Institute, Barts & The London School of Medicine & Dentistry, Queen Mary University of London, London, UK; 2 London Sportswise, London, UK; 3 Barts Health NHS Trust Physiotherapy Department, London, UK

**Keywords:** Exercise induced leg pain, Chronic exertional compartment syndrome, Medial tibial stress syndrome, Tibial stress fractures, Popliteal artery entrapment syndrome, Superficial peroneal nerve entrapment syndrome, Lumbar-sacral radiculopathy, McArdle’s syndrome, Myofascial tears and accessory/low-lying soleus muscle syndrome

## Abstract

**Background:**

Numerous conditions are grouped under the generic term exercise-induced leg pain (EILP), yet clear diagnostic guidelines are lacking. This scoping review was conducted to clarify the definition and diagnostic criteria of nine commonly occurring EILP conditions.

**Methods:**

Three online databases were searched from inception to April 2022 for any English language original manuscripts identifying, describing, or assessing the clinical presentation and diagnostic criteria of the nine most common conditions that cause EILP. We included manuscripts considering all adults with any reported diagnostic criteria for EILP in any setting. Methodological quality was assessed using the Mixed Method Appraisal tool. Condition definitions were identified and categorised during data charting. Twenty-five potential elements of the history, 24 symptoms, 41 physical signs, 21 investigative tools, and 26 overarching diagnostic criteria, were identified and coded as counts of recommendation per condition, alongside qualitative analysis of the clinical reasoning. Condition definitions were constructed with 11 standardised elements based on recent consensus exercises for other conditions.

**Results:**

One hundred nineteen retained manuscripts, of which 18 studied multiple conditions, had a median quality of 2/5. A combination of the history, pain location, symptoms, physical findings, and investigative modalities were fundamental to identify each sub-diagnosis alongside excluding differentials. The details differed markedly for each sub-diagnosis. Fifty-nine manuscripts included data on chronic exertional compartment syndrome (CECS) revealing exertional pain (83% history), dull aching pain (76% symptoms), absence of physical signs (78% physical findings) and elevated intercompartment pressure (93% investigative modality). Twenty-one manuscripts included data on medial tibial stress syndrome (MTSS), revealing persistent pain upon discontinuation of activity (81% history), diffuse medial tibial pain (100% pain location), dull ache (86% symptoms), diffuse tenderness (95% physical findings) and MRI for exclusion of differentials (62% investigative modality). Similar analyses were performed for stress fractures (SF, *n* = 31), popliteal artery entrapment syndrome (PAES, *n* = 22), superficial peroneal nerve entrapment syndrome (SPNES, *n* = 15), lumbar radiculopathy (*n* = 7), accessory/low-lying soleus muscle syndrome (ALLSMS, *n* = 5), myofascial tears (*n* = 3), and McArdle’s syndrome (*n* = 2).

**Conclusion:**

Initial diagnostic frameworks and definitions have been developed for each condition of the nine most common conditions that cause EILP, suitable for clinical consideration and consensus confirmation.

**Supplementary Information:**

The online version contains supplementary material available at 10.1186/s13047-023-00680-6.

## Background

Exercise-induced leg pain (EILP) is an umbrella term to describe pain in the leg induced by physical activities. It is commonly seen in young active individuals involved in competitive or endurance sports (including running, football, rugby, and dancing). The incidence of EILP among athletes has been reported to be between 12.8% and 82.4% [1–4]; however, it has not been reported for the general population [1–3]. The common element of EILP is a history of activity-induced leg pain, which commonly increases until activity cessation [5, 6]. In some cases, pain is also experienced at rest and manageable during activity. Apart from pain, other symptoms can include burning, cramping, swelling, muscle weakness, fatigue, malaise, paraesthesia, numbness, poikilothermia, and tightness [2, 7, 8].

There are more than 40 conditions (Additional file 1) that fall into the generic category of EILP, which requires clinicians to be vigilant when diagnosing patients presenting with exercise-related leg pain [2, 4–6, 9, 10]. The most common EILP entities fall into one of the following subdivisions [5]: (a) muscular origin pain, such as chronic exertional compartment syndrome (CECS) and myofascial tears; (b) pain of bony origin, such as medial tibial stress syndrome (MTSS) and tibial stress fracture (TSF); (c) pain due to nerve entrapment or compression, such as superficial peroneal nerve entrapment syndrome (SPNES); (d) radicular pain or radiculopathy; (e) pain of vascular origin due to temporary compression of either an artery or a vein, such as popliteal artery entrapment syndrome (PAES); (f) muscle disorders, such as McArdle’s syndrome; and (g) accessory or low-lying soleus muscle syndrome (ALLSMS) (also known as ASM) [2, 4–6, 9, 10]. These nine specific EILP conditions (CECS, MTSS, tibial SF, SPNES, myofascial tears, radiculopathy, PAES, McArdle syndrome, & ALLSMS) were the focus of this scoping review.

A detailed and methodical clinical history, knowledge of the conditions, appropriate examination and anatomical knowledge are regarded as key to reaching an appropriate diagnosis, while remaining aware that some of the conditions have overlapping signs and symptoms.

Because of coexisting conditions, a lack of consensus on the diagnostic guidelines and limited diagnostic test accuracy, EILP subdiagnoses cannot be clearly established in many cases [5, 10]. Clinical examination or physical findings are variably helpful, with the exception of nerve- or bone-related pathology. Investigations or further investigative modalities are often essential to confirm the diagnosis [10]. It is essential to be able to differentiate between the causes of EILP among patients to reach the most appropriate diagnosis to construct an appropriate treatment and management plan [5, 10].

There is a copious and varied body of literature describing EILP-related conditions and their clinical presentation; however, there is limited guidance facilitating differentiation between even the more common conditions. This variation contributes to diagnostic confusion, variable care, and suboptimal outcomes. Therefore, the aim of this scoping review was to collate and evaluate the published diagnostic criteria of the nine most commonly occurring EILP conditions to provide clear guidance about differential diagnosis.

Following an introductory literature search [11] and discussions between the authors, the research question—‘What are the diagnostic criteria for patients with EILP?’—was formulated to reflect the scoping review population, concept, and context.

## Methods

This scoping review was conducted and reported according to the Preferred Reporting Items for Systematic Reviews and Meta-Analyses Extension for Scoping Reviews (PRISMA-ScR) [12]. The methodological framework of this review is based on Arksey and O’Malley’s approach with modifications recommended by Levac et al., as well as the work of the Joanna Briggs Institute and previously published relevant scoping reviews [13–17]. The completed PRISMA-ScR Checklist is available (See Additional file 2) [12]*.*

A scoping review was the most appropriate methodological approach to achieve this aim due to the iterative, exploratory methodology the topic needed [18–20] A scoping review was also viewed as ideal to clarify key concepts, characterise findings from the diverse literature and outline the range of available evidence [11, 14, 15].

### Protocol and registration

The protocol was registered as an open-ended registration with Open Science Framework (OSF) (https://doi.org/10.17605/OSF.IO/4K27B) [21]. The plan was modified after searching three databases (PubMed, Embase, Scopus), and the team decided not to search five databases, as we found three conclusive databases. Therefore, the protocol was modified on August 2022 (https://doi.org/10.17605/OSF.IO/DBPJE) [22].

### Eligibility criteria

﻿ We included manuscripts considering adult people of any gender that contained a description of EILP diagnostic criteria in any format at any consultation in all health care settings. However, the diagnostic criteria did not need to be the focus of the manuscript to be included, i.e., if the recruitment involved detail of how a condition was screened for, or if in the introduction of a systematic review it was described, then that paper was included. There was no limitation on manuscript type. See Table 1 for the full inclusion and exclusion criteria.

**Table 1:** Eligibility criteria

Inclusion criteria	Exclusion criteria
Human participants	Animal models
English language	Not English language
Adult participants (18–60)	Children or elderly participants
Description of clinical diagnosis of EILP	Systemic diseases causing leg pain
Manuscripts looking at diagnosis, assessment, and clinical presentation	Manuscripts containing only a description of therapeutic approaches

### Information sources and search

﻿Three online databases were searched from inception to April 2022 (i.e., PubMed, Embase, Scopus) for relevant manuscripts. The keywords and constructs used to execute each search systematically were determined based on a preliminary search [11, 23] of the existing relevant reviews [4, 5, 10, 24, 25] and in consultation with the team members. A list of search terms (conditions, exercise, anatomical location, outcomes, and exclusions) is provided in Table 2, and the full search strategy for each database is available (see Additional file 3)*.* A decision was made to limit searches to the English language, guided and managed by the first author, and organised using the reference management software EndNote 20.

**Table 2:** Search terms

Search terms		
**Construct**		**Keywords**
Conditions	AND	“shin splints” OR EILP OR “exercise induced leg pain” OR “compartment syndrome” OR “chronic exertional compartment syndrome” OR “exertional compartment syndrome” OR CECS OR “anterior compartment syndrome” OR “posterior compartment syndrome” OR “lateral compartment syndrome” OR “stress fracture” OR “tibial stress fracture” OR “fibular stress fracture” OR “stress syndrome” OR “Medial tibial stress syndrome” OR “tibial stress syndrome” OR “tibial stress injury” OR MTSS OR “stress injury” OR “radicular leg” OR “leg radiculopathy” OR “lumbar radiculopathy” OR “lumbar radicular” OR “nerve entrapment syndrome” OR “neuropathy” OR “peroneal neuropathy” OR “superficial peroneal neuropathy” OR “arterial entrapment syndrome” OR “popliteal artery entrapment syndrome” OR (exercise pain) OR “Myopathy” OR “Glycogen Storage Disease Type V” OR “McArdle disease” OR “McArdle syndrome"
Exercise	AND	Exercise* OR active* OR sport* OR athlete* OR train* OR exertion OR practice* OR physical
Anatomical location	AND	Leg OR compartment* OR calf OR shin* OR “lower limb” OR tibia* OR fibula* OR fascia
Outcomes	AND	Diagnoses* OR “differential diagnosis” OR presentation OR “Clinical presentation” OR detection OR “clinical reasoning” OR findings
Exclusions	NOT	In title: acute OR disease OR diabetes* OR foot OR feet OR thigh OR shoulder OR arm OR hand OR wrist OR back OR Raynaud’s OR cervical radiculopathy

### Selection of sources of evidence

﻿After deduplication using EndNote 20, two levels of screening were conducted. The titles and corresponding abstracts of all returned manuscripts were evaluated against the eligibility criteria using online Rayyan [26]. Second, full-text copies of all manuscripts included at the title and abstract screening stage were retrieved and screened to determine the initial study selection. Both stages were performed independently by three of the authors (FB, KG, CM), with each paper being screened by two authors. Any disagreement over the eligibility of a particular manuscript was resolved through discussion among the team to determine final manuscript selection meeting the eligibility criteria. If an agreement was not reached, a fourth author was consulted. Further searching of reference lists of the included full-text manuscripts and citations of these were explored to identify additional relevant manuscripts not returned in the primary search and to ensure that the search strategy was capturing relevant manuscripts [12–14].

### Data charting

A customised coded Excel sheet was designed to collate the charted data in a consistent format (Additional file 4). Following an initial discussion and brief exploration of the returned manuscripts, the main headers were chosen: i) definition of the condition, ii) anatomical location, iii) prevalence and incidence, iv) history, v) symptoms, vi) physical findings, vii) aggravating activity, viii) diagnostic criteria used within the literature, ix) investigative tools used, and x) therapeutic approaches undertaken and their efficacy. Within each of these main headings, subheadings were iteratively developed to classify the approaches described in each manuscript. Details of the population demographics, such as age and sex, were included wherever reported, alongside year of publication, author name, and study design [5, 6]. Three authors charted data from the first 10 manuscripts independently to determine whether their approach to the charting process was consistent [13, 27]. The full data charting process was conducted by one author (FB) for consistency, while two other authors (KG and CM) undertook approximately 50% each, so that data from each paper were checked by at least two reviewers.

### Quality assessment

The methodological quality of retained manuscripts was evaluated by three authors (FB, KG and CM) using the Mixed Methods Appraisal Tool (MMAT) 2018 Version [28]. The MMAT measures five various methodological classifications, enabling use across different study designs, and is valid, reliable, and efficient [28, 29]. Inconsistencies in the MMAT scoring between the authors were resolved by agreement between them and accessing another author as needed.

### Results synthesis

#### Condition definitions

In addition to the synthesis of reported diagnostic approaches, definitions for the nine conditions were recorded from each included manuscript, and a single definition for each was formulated by first identifying the most common definitions and their sources. The literature was searched to construct operational definitions to provide in the review. The content was then cross-referenced to a standardised structure derived from analysis of all definitions and consideration of previous reports of consensus definitions for chronic conditions [30]. The domains included [condition name], causing [selection of symptoms/impairments including frequency and severity], for at least [minimum duration], at [location], resulting in [disability], in [population], for [x duration], when [exercise relationship], because [proposed mechanism], confirmed by [y and z test(s)], and [other conditions] particularly need to be excluded.

#### Data coding and synthesis of main domains

Based on discussions among the study team and reviewing relevant conceptual and methodological articles [15–17, 31, 32], data were coded.

The five main domains identified initially within the data charting process were history, symptoms, physical findings, investigations and overarching diagnostic criteria. The number of manuscripts that reported the use of an approach within the diagnosis was coded and counted under each subdomain element. Synthesis of reporting across manuscripts was undertaken for each of the nine EILP-related conditions and is presented as the percentage of manuscripts reporting a diagnosis approach within the number of manuscripts reporting a diagnosis for the specific EILP diagnosis. As such, the total number of manuscripts differed across injuries, and a single manuscript might include data on multiple conditions.

The synthesised data were presented in condition-specific graphs, which show the proportion of manuscripts reporting a given diagnostic criterion. Data were combined for the nine conditions into a separate figure for each of the five main domains charted (all details are available in Additional file 4).

## Results

### Selection of sources of evidence

**Fig. 1:**
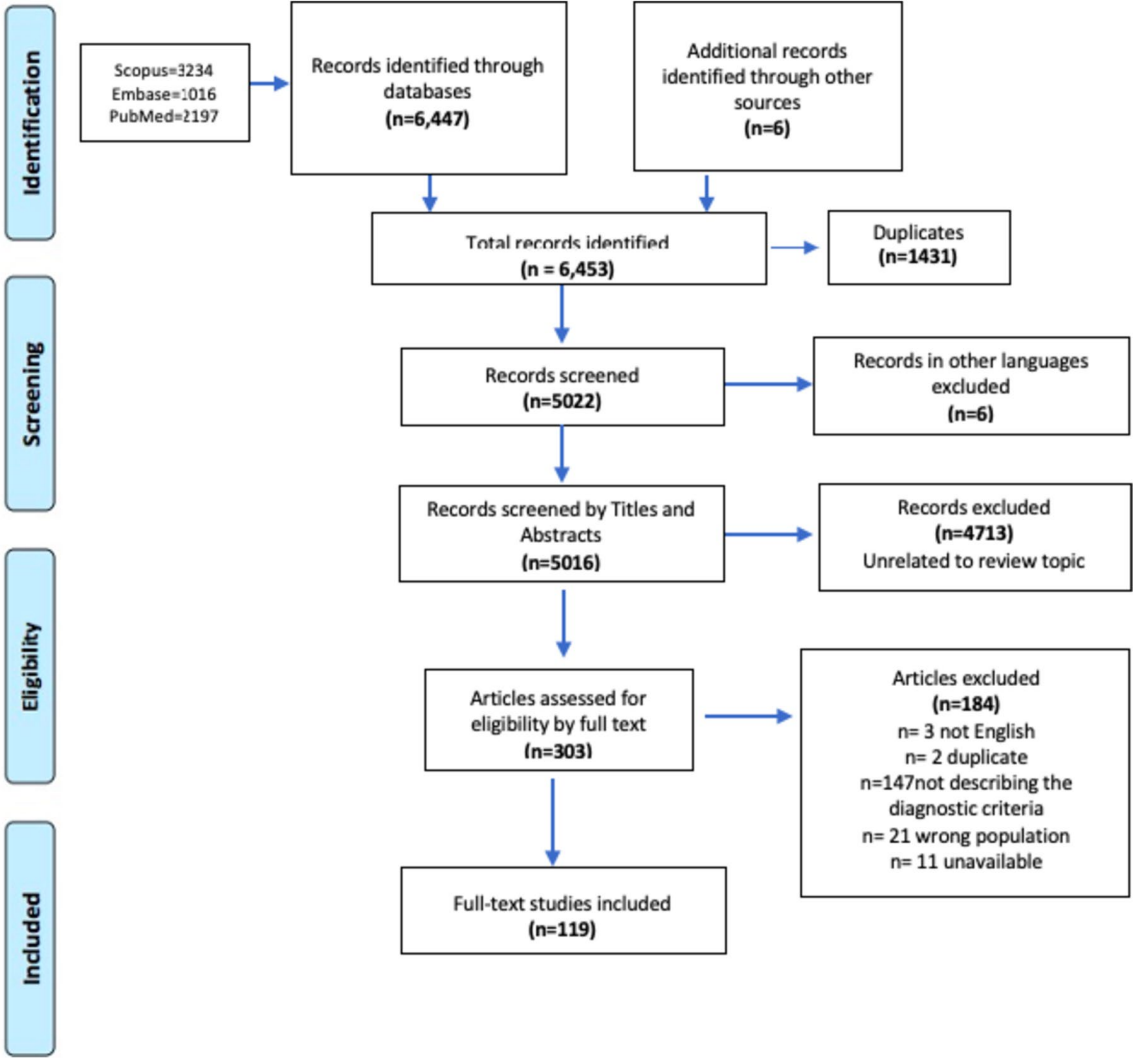
Preferred Reporting Items for Systematic reviews and Meta-Analyses extension for Scoping Reviews (PRISMA-ScR) flow chart

﻿The search yielded 5022 manuscripts after deduplication. After the title and abstract screening stage, 303 manuscripts were reviewed in full. Of these 303 manuscripts, 119 were retained, and 184 were excluded because they did not meet the eligibility criteria as outlined in Fig. 1*.* Eleven of the full text articles were not available through online referral sources, and the authors did not respond to requests to share the full text.

### Characteristics of sources of evidence

A summary of the types of manuscripts included in this review is shown in Table 3. Noncomparative studies including opinion pieces/narrative reviews accounted for 45.4% of the included manuscripts [4, 6, 33–84], case reports (22%) [7, 85–109], and case series studies (17%) [110–129]. Five percent of the included manuscripts were systematic reviews [5, 130–134]. Case control studies accounted for 2.5% [135–137], as well as retrospective case series studies (2.5%) [24, 138, 139]. Cross-sectional studies accounted for 2.5% [140–142]. Experimental quantitative trials accounted for 2.5% (randomised and nonrandomised controlled trials) [143–145]. In all, 0.8% of the manuscripts were observational cohort studies [146].

**Table 3:** Type of manuscripts included and evidence level of different study types

Type of manuscripts	Number	Evidence level
Opinion piece/narrative review	54 (45.4%)	5
Case reports	26 (22%)	5
Case series	20 (17%)	4
Systematic reviews	6 (5%)	2
Case‒control studies	3 (2.5%)	4
Retrospective case series	3 (2.5%)	4
Cross-sectional	3 (2.5%)	4
Randomised controlled trials (RCT)	2 (1.7%)	2
Nonrandomised controlled trials (non-RCT)	1 (0.8%)	3
Cohort studies	1 (0.8%)	2
	**119 total**	**Median 4**

### ﻿Quality assessment

The median quality of the included manuscripts was 0.4, with a mean of 2.01 (IQR = 3/0–3) using the MMAT (for details, see Additional file 5)*.* Most of the nonsystematic reviews and opinion pieces/narrative reviews were rated as poor to moderate quality (0–1 scores), while case-series studies, case control studies, and experimental quantitative trials had higher MMAT scores (3–4 scores).

### Data extraction

Additional file 4 presents the completed data charting table and the individual charted data from each resource of evidence.

### Synthesis of results

#### Condition definitions

Synthesised definitions of the nine most common conditions causing EILP are provided in Table 4***.*** Not all included manuscripts provide a specific definition of the involved condition, with 101 manuscripts describing only one condition, while 18 described more than one condition. Additionally, none of the included manuscripts defined myofascial tears, and the definition was synthesised from other literature resources. The purpose of this work was to produce definitions based on the current literature to represent current thinking but in a format suitable to inform later consensus work on the definitions.

**Table 4:** Definitions of the nine most common EILP conditions

Condition	Synthesised definition and key early sources
**Chronic exertional compartment syndrome**	… is defined as increased pressure in the lower leg compartment causing severe pain, tightness, and paraesthesia which in severe cases that would occur every time sufferers exercise for at least 6–12 months. The anterior compartment is most affected and symptoms usually bilateral, affecting the ability to do competitive sports. It is more common in young males. Symptoms may persist for months to years and usually build during activity until they must stop, pain then settle with rest, recurring on return to activity. The mechanism is tightness in the affected compartment due to an increase in intramuscular pressure, resulting in a lack of perfusion and altered physiology. CECS diagnosis is confirmed by measurement of dynamic intracompartment pressure. SPNES and MTSS need to be excluded.
	22 definitions reported in 59 (37%) relevant publications 2 common partial definitions, first reported in [147, 148]
**Medial tibial stress syndrome**	… is defined as periostitis causing diffuse pain for months to years at the distal posteromedial border of the tibia, which can be unilateral or bilateral, and lead to compromised ability to exercise. It is more common in young active individuals involved in endurance activity (runners, dancers, and military recruits), and can last for a few hours to days per episode. The mechanism is subcutaneous periostitis associated with altered biomechanics of the lower limb, training errors and increased training intensity. MTSS diagnosis is confirmed by clinical history, site of pain, palpation, MRI scan and bone scan. Stress fracture and CECS of the deep posterior compartment need to be excluded.
	11 definitions reported in 21 (52%) relevant publications 2 common definitions, first reported in [132, 146]
**Tibial stress fracture**	… is defined as cortical or full bone fracture causing localised excruciating pain both at rest and with weight-bearing activity with nocturnal pain for days to few weeks. This occurs on a daily basis until it heals, typically at the middle to lower one-third of the tibia most commonly but can occur anywhere. The acute pain can cause gross disability and usually affect young age group 10–30 years of age and it heals in a minimum of 6–8 weeks with immobilisation. The mechanism is an imbalance between osteoblast and osteoclast activity leading to bone breakage due to overtraining, overuse, and repeated overloading. Tibial stress fracture diagnosis is confirmed by localised tenderness, plus positive hop and/or fulcrum test. Bone tumours and frank fracture need to be excluded.
	10 definitions reported in 31 (32%) relevant publications 2 common definitions, first reported in [48, 149]
**Superficial peroneal nerve entrapment syndrome**	… is defined as mechanical compression of the superficial peroneal nerve causing moderate to severe pain, paraesthesia, numbness, and the feeling of a ‘restless leg’ for few months to years at unilateral anterior compartment of the leg. It is more common in young active adults and each episode can last for minutes to an hour. The symptoms occur with activity and are relieved with a variable period of rest. The mechanism is scarring/entrapment around the opening of the fascia where the nerve becomes a cutaneous sensory nerve supplying the dorsum of the foot. SPNES diagnosis is confirmed by diagnostic local anaesthetic test. CECS needs to be excluded.
	3 definitions reported in 15 (20%) relevant publications 2 common definitions, first reported in [150, 151]
**Myofascial tear**	… is defined as a single event causing a tear within the myofascial causing pain with activity as the main symptom and can be severely disabling. Located at the interface between the aponeurosis and the fibre or muscle fasciculus (and its corresponding perimysium). It is more common in young active adults and can be acute or chronic. In chronic cases, pain occurs with activity and is relieved with rest. The mechanism is the tendon or aponeurosis affected either focally by small muscle fibre tears or by major tears that produce a muscle gap but not a tendinous gap. Myofascial tears diagnosis is confirmed by dynamic ultrasound or, more reliably for deep tears, MRI. Muscle tears, haematoma, radiculopathy, and sural nerve entrapment syndrome need to be excluded.
	0 definition reported in 3 (0%) relevant publications 1 common definition, first reported in [152, 153]
**Lumbar radiculopathy**	… is defined as mechanical compression of a nerve root at the level of the spinal cord as it exits the foramen or lateral recess causing sharp pain … radiates down the legs, paraesthesia, numbness, spontaneous cramp, lack of power, fatigue, and tiredness in one or both legs during activity and at rest for months to years. It usually affects the posterior dermatomes and myotomes of the legs but can occur in other areas leading to restricted movement, disturbed sleep, and altered ability to exercise. It is more common in males 30–50 years old. The mechanism is mechanical compression of a nerve root at the level of the spinal cord as it exits the foramen or lateral recess. Lumbar radiculopathy diagnosis is confirmed by lumbar-sacral MRI scan and sometimes EMG/nerve conduction study. Gluteal and piriformis syndromes, myopathy, and in some cases unusual and uncommon CECS and PAES need to be excluded.
	3 definitions reported in 7 (42%) relevant publications 1 common definition, first reported in [154]
**Popliteal artery entrapment syndrome**	… is defined as arterial insufficiency in the affected limb which arises with entrapment of the artery, commonly giving leg symptoms with exertion causes pain, poikilothermia along with tightness, paraesthesia, and numbness can also occur for weeks to three months at the superficial posterior compartment, and it is usually unilateral, leading to intermittent claudication and temperature changes. It is more common in young active runners and each episode can lasts for few minutes. The mechanism is an abnormal relationship between the popliteal artery and the surrounding myofascial structures in the popliteal fossa. PAES diagnosis is confirmed by MRI, MRA, CT, Angiography, Duplex ultrasound scan. Radiculopathy, soleus sling syndrome, and CECS affecting the superficial posterior compartment need to be excluded.
	11 definitions reported in 22 (50%) relevant publications 1 common definitions, first reported in [80]
**McArdle’s syndrome**	… is defined as autosomal recessive metabolic myopathy causes pain, tightness, swelling, malaise, and lethargy for years at multiple muscle compartments of the upper and lower limbs, leading to fixed weakness, malaise, fatigue, and tiredness. It is more common in young active population; it is a long-term condition that occurs during exercise. The mechanism is an autosomal recessive metabolic myopathy causing exercise-induced rhabdomyolysis due to a deficiency of muscle phosphorylase. McArdle’s syndrome diagnosis is confirmed by resting creatine kinase (CK) followed by 3 consecutive postexercise CK and muscle biopsy. Other medical myopathies and CECS need to be excluded.
	1 definition reported in 2 (50%) relevant publications 1 common definition, first reported in [57]
**Accessory/low-lying soleus muscle syndrome**	… is defined as a space occupying mass which can cause nerve compression which mimics tarsal tunnel syndrome and can also cause increase in the intercompartmental pressure mimicking CECS causes soft tissue swelling that may be painful during physical activity for few months to years. Other symptoms that may be attributed to impingement on neurovascular structures and paraesthesia and numbness affecting the plantar aspect of the foot. It occurs at the superficial posterior compartment and can be unilateral or bilateral and more common in young active adults. The mechanism is a rare supernumerary anatomical variant. ALLSMS diagnosis is confirmed by MRI and ultrasound scans. CECS affecting the superficial posterior compartment, PAES, and radiculopathy need to be excluded.
	1 definition reported in 5 (20%) relevant publications 2 common definitions, first reported in [155, 156]

#### Synthesis of main domains and diagnostic criteria

The results synthesis displays the frequency of each diagnostic element within the main domains for all conditions individually. The elements within the domains with the highest frequency for a certain condition are the ones reported the most within the included manuscripts; lower frequencies indicate that the element is less frequently reported within the manuscripts for diagnosing that condition.

Twenty-five potential subdomains/elements for history, 24 for symptoms, 41 for physical signs, and 21 for investigative tools were identified within the retained manuscripts. The fifth main domain identified was the overarching diagnostic criteria, some of which reproduced data in the other four main domains but had been highlighted by manuscript authors.

##### History domain

**Fig. 2:**
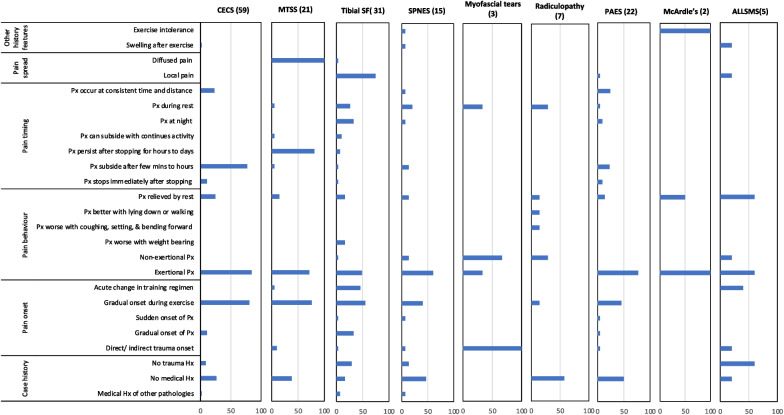
This bar chart details the frequency with which each of the 25 most common elements of the patient’s history were reported as being relevant to each condition. Column headers indicate the condition and the number of relevant manuscripts in brackets. CECS = chronic exertional compartment syndrome, MTSS = medial tibial stress syndrome, tibial SF = tibial stress fracture, SPNES = superficial peroneal nerve entrapment syndrome, PAES = popliteal artery entrapment syndrome, ALLSMS = accessory/low-lying soleus muscle syndrome. Px = Pain. Hx = History

The most reported elements of the medical history indicating *CECS* were exertional pain, gradual onset during the exercise, and the relief of pain within a few minutes after discontinuing the activity (frequency = 83%, 80%, and 76% of papers, respectively), as shown in Fig. 2*.* Pain relief soon after activity cessation appeared to be specific to *CECS* cases, with it only minimally being reported as a diagnostic tool for *PAES, SPNES, tibial SF and MTSS* (23%, 13%, 3%, and 5%, respectively). The most reported history elements for *MTSS* were diffuse pain spread within the distal two-thirds of the tibia, pain persistence from a few hours to days, and gradual symptom onset during activity (100%, 81%, 76%, respectively). The first two elements were specific to the *MTSS* presentation pattern. However, for *tibial stress fracture*, local pain spread (< 5 cm), gradual onset of pain during exercise, and exertional pain were reported to be the history pattern in 74%, 55%, and 48% of patients, respectively. Localised pain was specific to *tibial stress fractures* (74%).

There were no history elements unique for SPNES and *radiculopathy*, as the main reported elements for *SPNES* were exertional pain, no medical history related, and gradual pain onset (60%, 47%, 40%, respectively), while the most common elements for *radiculopathy* were no prior medical history, nonexertional pain, and pain during rest (57%, 28.6%, 28.6%, respectively). For *PAES,* the typical elements were exertional pain type, absence of related medical history, and gradual onset during activity (77%, 50%, and 46%, respectively). The most frequent history elements for *myofascial tears* were direct/indirect trauma, nonexertional pain, and pain at rest (100%, 67%, 33%, respectively), with the history of trauma being particular to this condition. Cases of *McArdle’s* syndrome were found to have a well-defined pattern; however, the elements were not particular to this syndrome, including exercise intolerance, exertional pain and pain relieved by rest (100%, 100%, and 50%, respectively). *ALLSMS* history was unclear, with the reported elements of swelling after exercising, pain being relieved by rest and exertional pain ranging from 20%-60%.

##### Symptoms domain

**Fig. 3:**
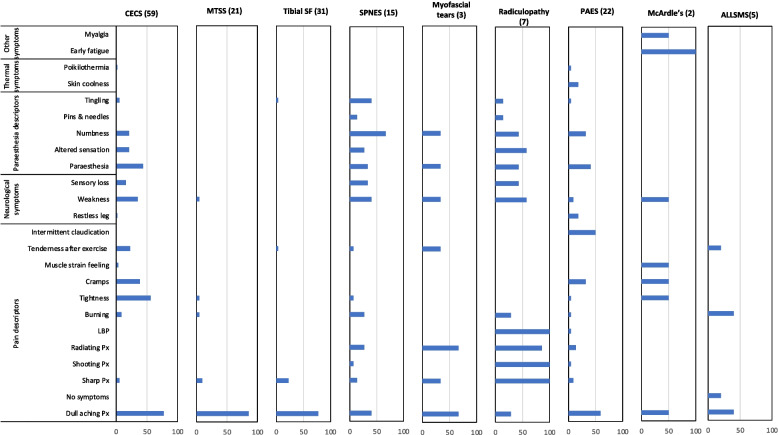
This bar chart details the frequency with which each of the 24 most common elements of the patient’s symptoms were reported as being relevant to each condition. Column headers indicate the condition and the number of relevant manuscripts in brackets. CECS = chronic exertional compartment syndrome, MTSS = medial tibial stress syndrome, tibial SF = tibial stress fracture, SPNES = superficial peroneal nerve entrapment syndrome, PAES = popliteal artery entrapment syndrome, ALLSMS = accessory/low-lying soleus muscle syndrome. Px = Pain. LBP = low back pain

The most reported symptoms are shown in Fig. 3, with a dull ache, tightness, and paraesthesia being the main symptoms for *CECS* (76%, 54%, and 44% respectively). A dull ache was also the main symptom reported for *MTSS* and *tibial stress fracture* (86% and 77%, respectively). However, *tibial stress fracture* also presented with sharp pain in some (23%) patients. Neurological symptoms, including numbness, tingling, and weakness (67%, 40%, 40% respectively), were the most common indications for *SPNES*. *Myofascial tears* can present with different types of pain (dull achy 67%, radiating 67%, and sharp 33%), while other commonly reported symptoms include altered sensation and tenderness after exercising (both 33%). The presence of low back pain (LBP), sharp pain, and radiating pain could be an indicator of *radiculopathy*, all of which returned 100%. The highly reported symptoms for *PAES* were dull ache, intermittent claudication, and paraesthesia (59%, 50%, 41%, respectively). LBP and intermittent claudication (100% and 50%, respectively) were specific symptoms for *radiculopathy* and *PAES,* respectively. The most specific reported symptoms for *McArdle’s syndrome* were early fatigue, myalgia, and weakness (100%, 50%, and 50%, respectively). *ALLSMS* reports included dull aching, burning, and tenderness (40%, 40%, 20%, respectively); however, it could also present with no symptoms (20%).

##### Findings of the physical examination procedures domain

**Fig. 4:**
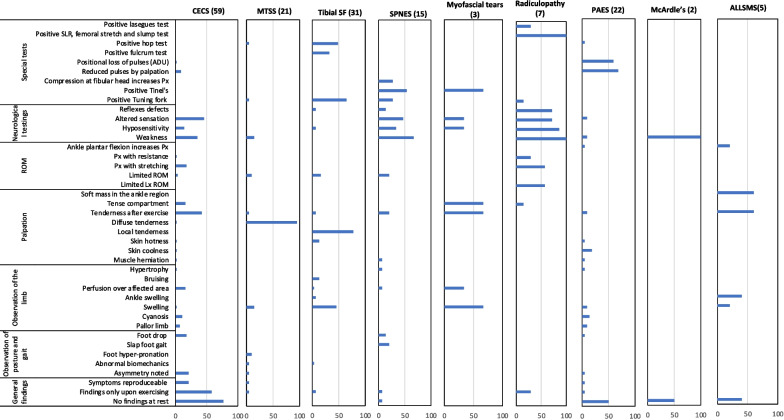
This bar chart details the frequency with which each of the 41 most common elements of the patient’s physical findings were reported as being relevant to each condition. Column headers indicate the condition and the number of relevant manuscripts in brackets. CECS = chronic exertional compartment syndrome, MTSS = medial tibial stress syndrome, tibial SF = tibial stress fracture, SPNES = superficial peroneal nerve entrapment syndrome, PAES = popliteal artery entrapment syndrome, ALLSMS = accessory/low-lying soleus muscle syndrome. Px = Pain. ROM = Range of motion

Physical examination for patients with EILP was typically categorised as either observation, palpation, range of motion (ROM), neurological testing, or special tests. Therefore, Fig. 4 is presented to illustrate the most common physical findings used to establish a diagnosis. Interestingly, the absence of physical findings at rest was most reported for *CECS* (78%), followed by altered sensation and tenderness after exercising (46% and 41%, respectively). Diffuse tenderness on palpation presented in more than 95% of *MTSS* literature but absent in all others was specific for *MTSS*. Other findings, such as weakness and swelling, were reported with a lower frequency for *MTSS* (both 14.3%). The presence of local tenderness, a positive tuning fork and a hopping test were reported as being indicative of a *tibial stress fracture* (77%, 65%, and 48%, respectively). The main reported physical findings for *SPNES* were weakness, positive Tinel’s test, and altered sensation (67%, 53%, and 47%, respectively). The most reported findings for *radiculopathy* were weakness, positive slump test, femoral stretch, and straight leg raise, all of which returned 100%. Signs of swelling, tenderness after exercise and tense muscle compartment (67%) were the most common findings of *myofascial tears*. The most reported findings for *PAES* were reduced pulses, positional loss of pulses using arterial Doppler ultrasonography, and the absence of signs while resting (68%, 59%, 50%, respectively). Weakness was the only physical finding reported for *McArdle’s syndrome*, as well as the absence of signs during rest (100% and 50%, respectively). The appearance of a soft tissue mass in the ankle region, alongside tenderness after exercising, and pain during ankle plantar flexion (60%, 60%, 20%, respectively) were the main findings indicating *ALLSMS*.

##### Further investigations domain

**Fig. 5:**
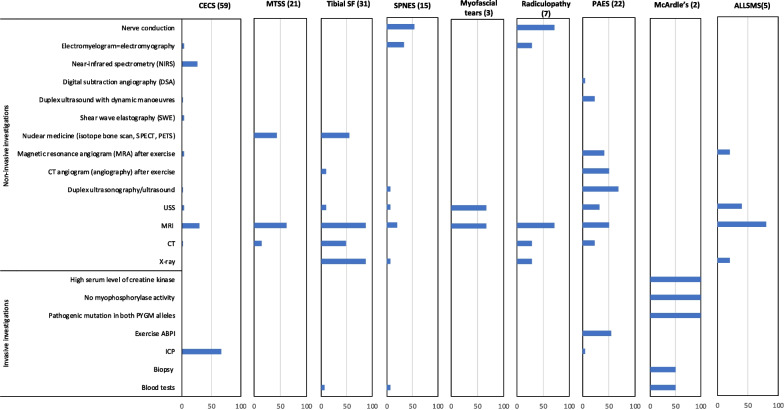
This bar chart details the frequency with which each of the 21 most common elements of the further investigation’s modalities were reported as being relevant to each condition. Column headers indicate the condition and the number of relevant manuscripts in brackets. CECS = chronic exertional compartment syndrome, MTSS = medial tibial stress syndrome, tibial SF = tibial stress fracture, SPNES = superficial peroneal nerve entrapment syndrome, PAES = popliteal artery entrapment syndrome, ALLSMS = accessory/low-lying soleus muscle syndrome. CT = computed tomography scan. USS = Ultrasound scan. MRI = Magnetic resonance imaging. ABPI = Ankle brachial pressure index. ICP = Dynamic intracompartment pressure

Further investigative modalities, as displayed in Fig. 5, were used chiefly as the concluding step in the diagnostic process as positive or negative confirmation of the clinical diagnosis. MRI was frequently used for all conditions to confirm a particular diagnosis (*ALLSMS* 80%, *tibial SF* 78%, *radiculopathy* 71%, *myofascial tears* 66%, *PAES* 50%, *SPNES* 20%), or to rule out other pathologies (*MTSS* 62%, *CECS* 31%), except for the absence of its use as a diagnostic tool for *McArdle’s*. Conversely, *McArdle’s* diagnosis was reported to be confirmed by more invasive tastings, i.e., genetic screening, blood test, and biopsy (100%, 50%, and 50%, respectively). The use of dynamic intracompartment pressure (ICP) tests returned a 66% frequency for *CECS*, with low to no returns for the others. Further measurement of the ankle brachial pressure index (ABPI) was only used to confirm cases of *PAES* (31%) alongside the use of some imaging modalities (ultrasonography 68%, MRI 50%, CT angiogram 50%) after provoking symptoms with exercises and manoeuvres as determined by the history. The diagnostic process of *stress fractures* has been reported to be based on the use of imaging modalities, such as X-ray, MRI, and bone scan (87%, 87%, 54%, respectively). In addition, X-ray can be a useful indication of the presence of *ALLSMS* (20%), and ultrasound scans can reveal *myofascial tears* (66%). Moreover, nerve conduction studies could assist in the diagnosis of *radiculopathy* and *SPNES* (71% and 53%, respectively).

### Diagnostic criteria

**Fig. 6:**
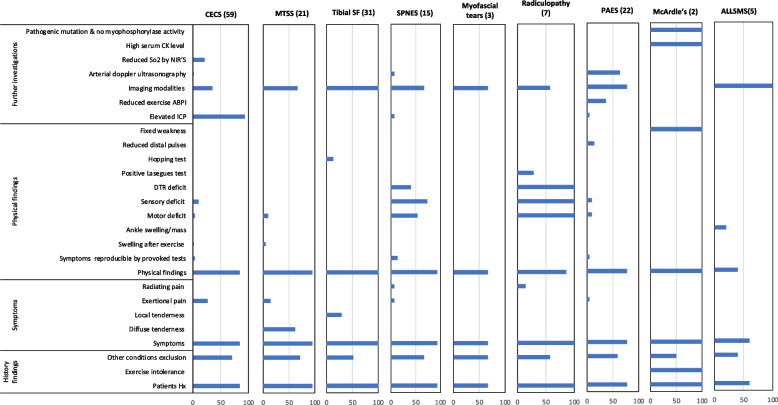
This bar chart details the frequency with which each of the 26 most common diagnostic criteria were reported as being relevant to each condition. Column headers indicate the condition and the number of relevant manuscripts in brackets.CECS = chronic exertional compartment syndrome, MTSS = medial tibial stress syndrome, tibial SF = tibial stress fracture, SPNES = superficial peroneal nerve entrapment syndrome, PAES = popliteal artery entrapment syndrome, ALLSMS = accessory/low-lying soleus muscle syndrome. CK = Creatine kinase. So2 = Oxygen saturation. NIRS = Near-infrared spectrometry. ABPI = Ankle brachial pressure index. ICP = Dynamic intracompartment pressure. DTR = Deep tendon reflex. Hx = History

The overall diagnostic criteria for these nine conditions are presented in Fig. 6, which represents the frequency of each of the 26 most common diagnostic criteria reported for each of the conditions, including those reported in the previous four domains. In other words, the resultant overarching diagnostic criteria for these nine conditions consist of 24 criteria reported in the included manuscripts as individual criteria plus the other four domain elements (history, symptoms, physical findings, further investigations).

## Discussion

This comprehensive scoping review details the definitions and key diagnostic criteria for the nine most common conditions that cause EILP based on the literature. The findings are collated and evaluated by condition.

Single definitions for each of the nine conditions were synthesised by identifying the most common definitions and their sources within the literature and then checked according to a standardised domain. These are qualitative outputs of the data synthesis, using criteria from the clinical case definition of post-COVID-19 syndrome in a previous Delphi consensus [30]. A Delphi study of healthcare professionals and patients was conducted in a three-stage process to define core health-related domains for tendinopathy, and they found that nine core domains for tendon research should guide the reporting of outcomes in clinical trials [157, 158]. This imposes the importance of having clear definitions domains for such clinical conditions. The diversity in definition between manuscripts was notable, with some manuscripts not providing any definition and most lacking important details (including frequency, least, duration, mechanism) that would enable comparison between studies and evidence synthesis. By combining all of these in a standardised structure, we aim to provide a clear foundation for promoting discussions in the future and use of these synthesised definitions for further consensus processes.

*The diagnosis of CECS* revolved primarily around the pain pattern of the relief within a few minutes after discontinuing the activity, the absence of physical findings when not exercising and invasive ICP measuring procedure. Elevated ICP is proposed to be a diagnostic tool for CECS within the results. This was accepted by Pedowitz et al., who demonstrated elevated values in the affected compartments and a resting pressure greater than 15 mmHg and 5 min postexercise pressures greater than 20 mmHg [139]. However, current studies outlining these criteria have no high-level evidence. Therefore, current ICP pressure criteria for CECS diagnosis are consequently unreliable, and emphasis should remain on a clear history [81, 159], which was also presented within this review [81, 159].

*The diagnosis of MTSS* was based mainly on a history of persistent, cumulative pain that does not immediately cease after stopping activity and may persist between a few hours and a few days, as reported in 81% of the papers. This is accompanied by diffuse tenderness of the tibial periosteum during physical examination (95% reporting frequency). Imaging, mainly MRI, was also used, albeit recommended in only 62% of manuscripts to confirm MTSS in severe cases and to exclude other conditions [5, 140, 160–162].

*For tibial stress fracture,* diagnosis is first based on a history of gradual onset of focal pain during exercise, which matches the presence of localised pain on palpation and positive imaging findings featured in all manuscripts. Other specific tests have been used and reported to be useful in this review, for example, the hop test or fulcrum test. However, the hop test was found to be 100% sensitive and 45% specific for diagnosing medial tibial stress fracture, whereas the fulcrum test was not proposed to be statistically specific and sensitive for the diagnosis of medial tibial stress fracture [38, 110]. Therefore, the history, palpation and imaging modalities are consistent, and core elements are needed to detect stress fracture [38, 110, 163, 164].

*The key distinctive element for the diagnosis of PAES* was suspicion from the history of intermittent claudication type of pain, positional loss of pulse in the tibialis posterior artery during physical examination, and the absence of pain while resting. Sinha et al. identified that provocation Doppler ultrasonography, ankle brachial pressure index measurement, and MRI & MR angiography have 94% and 90% sensitivity for diagnosing PAES [165]. However, these were only stipulated in ~ 50% of the relevant manuscripts, suggesting that there is inconsistency of diagnostic approaches that merits further study.

*The diagnosis of SPNES* was based mainly on a history of nonspecific exertional neurological type of pain over the anterior compartment, accompanied by weakness, positive Tinel’s test, and altered sensation. Nerve conduction studies and electromyography were not found to be very useful unless pre- and postexercise symptom provocation tests were employed. MRI could be useful to demonstrate anatomical relations causing nerve compression [10, 166].

*Lumbar radiculopathy* diagnosis revolves primarily around the history of radiating pain, worsening of pain by coughing, bending, or sitting, neurological symptoms, and altered neurological findings during special testing, supporting the literature [154, 167]. However, this review found that nonexertional pain was relatively common in radiculopathy. This anomaly shows that the onset of pain may be important for radiculopathy diagnosis and that radicular pain can occur without exercise, unlike the other conditions, which are primarily exertional in nature, although pain may linger after exertion for a while in conditions such as MTSS. Radiculopathy diagnosis is mainly confirmed by imaging modalities, particularly MRI, as reported in ~ 70% of the relevant manuscripts.

*For myofascial tears,* the diagnostic criteria reported within this review are quite reflective of the literature (history of traumatic onset and sharp pain) [168, 169]. However, there was conspicuously little literature for this condition, despite it being a more frequent cause of EILP than other muscle/myofascial pathologies, which led to inconsistencies in these findings. This clearly indicates a lack of a diagnostic guide for the condition. Further research is required to establish a more reliable diagnostic approach for myofascial tears. This gap within the literature may clarify the unexpected dominant diagnostic tool for the condition according to the scoping review: imaging modalities. However, these factors play a role in the diagnosis of all conditions; therefore, they are not primary for the diagnostic procedure.

*The key distinctive element for the diagnosis of McArdle’s syndrome* is genetic PYGM testing and blood screening (including serum creatine kinase and genetic testing), giving it a clearly different diagnostic approach to the rest of the EILP conditions and is well represented in this review as expected. It was also characterised by early fatigue and myalgia due to genetic abnormalities. Lucia et al. reported that the diagnosis is usually confirmed by muscle biopsy, which shows a negative histochemical reaction for myophosphorylase and no myophosphorylase activity [57]. McArdle’s syndrome placed a large importance on further investigations for pathogenic mutations and the presence of certain symptoms and signs, including exercise intolerance and fixed weakness (all of which were returned within 100% of the literature).

*For ALLSMS*, diagnosis relied heavily on the absence of traumatic history, the lack of major presenting symptoms and the presence of anatomical variation on examination with (20%) return. Imaging modalities were recommended to confirm the diagnosis. This was a similar diagnostic approach to the literature, with physical findings revealing an extra muscle or low-lying muscle that can be clearly identified by imaging [102]. Accessory and low-lying soleus muscles are present as normal anatomical variations in many asymptomatic subjects. A significant increase in intensity and exercise workload, in some cases, may lead to pain and disability. These findings are also in agreement with the literature [170].

For each condition, a combination of key aspects of the history, physical findings, and further investigative modalities were fundamental to reach a diagnostic conclusion and formulate a diagnosis*.* This diagnostic framework will be useful for clinicians dealing with these conditions to improve differential diagnosis and reduce diagnostic variability. These findings must, however, be treated with caution, as study quality is low. Therefore, the diagnostic procedure will be easier when there are established diagnostic guidelines.

### Limitations of the literature

There was a paucity of literature for some of the included conditions (myofascial tears, McArdle’s syndrome, ALLSMS), as indicated by the low numbers of returns for these conditions. There was a lack of detailed diagnostic studies reporting sensitivity and specificity for any condition and a lack of longitudinal studies and trials, meaning that the strength of our conclusions is limited. Considering the level of evidence for the included manuscripts, the median is four, which indicates that most of the included manuscripts were of low quality. Additionally, there was a lack of consensus on definitions, which we hope our review can facilitate. Moreover, the prevalence of these conditions is not clear within the literature; therefore, there is a need for better epidemiological studies. Finally, the literature does not fully reflect clinical practice, as many patients present with an unclear picture; therefore, a consideration of how to deal with atypical presentations is an important consideration for future research that is not currently represented in the literature.

### Limitations of the review

There was only a partial critical appraisal (quality assessment, not risk of bias) of sources conducted due to the type of review. However, the mixed methods appraisal tool (MMAT) was used to assess the quality of the included manuscripts. Moreover, the authorship of the included manuscripts was not considered within this review, and this could be quite influential for the number of results due to the weight of each manuscript, especially where the same author has written multiple papers using the same diagnostic criteria. In addition, the domains we used for defining the conditions do not include the patient’s description and patient voice, and this may be worth considering in future work [157, 158]. Moreover, the data included in this review did not compare the evidence of different diagnostic modalities to assist in the diagnosis.

## Conclusion

In this scoping review, we developed definitions with a standardised structure and propose an initial diagnostic framework for nine common EILP sub-conditions. Patient history, anatomical symptom location, physical findings and investigative modalities represent crucial diagnostic elements. Many of the findings are consistent across the literature, but others are less so—highlighting both the inconsistencies between different clinical groups’ diagnostic approach to EILP and the need for efforts to build consensus. Notwithstanding the overlapping symptoms, primary patterns of presentation vary greatly between the conditions, and clinicians should be able to differentiate using the findings in this scoping review. All the conditions reported the necessity to exclude other differentials, reinforcing the view that EILP diagnosis is often one of exclusion. Adoption of this diagnostic framework and application of the associated definitions should help reduce care variability, improve EILP diagnosis and therefore improve management and outcome.

### Future research recommendations

1. Consensus studies on the resulting definitions and diagnostic criteria could be a potential for future research. We are hopeful that at the end of the current 4-phase study (SR is phase 1), we will have guidelines on diagnostic criteria for EILP.
2. Prevalence of the various conditions needs to be addressed.
3. Intra-compartment pressure measurement in supporting the diagnosis of CECS requires a particular study.
4. Use of dynamic MRI & MRA in the assessment of PAES needs evaluation.

### Supplementary Information

**Additional file 1.** Collection of EILP diagnosis since 1986.

**Additional file 2.** Preferred reporting items for systematic reviews and meta-analyses extension for scoping reviews (PRISMA-ScR) checklist. The completed PRISMA-ScR checklist.

**Additional file 3.** Full search strategy. The full search strategy used within each database.

**Additional file 4.** Developing a framework for the diagnostic criteria of patients with EILP scoping review data charting table. The data charting table for this review.

**Additional file 5.** Developing a framework for the diagnostic criteria of patients with EILP scoping review mixed method appraisal tool. The mixed method appraisal tool (MMAT) scoring for this review.
