# Supplementary material for: Developing a diagnostic framework for patients presenting with Exercise Induced Leg Pain (EILP): a scoping review
Source: J Foot Ankle Res. 2023 Nov 21;16:82. doi: 10.1186/s13047-023-00680-6 (PMC10662794; doi:10.1186/s13047-023-00680-6)
Supplement: Supplementary file 1 — Additional file 1. Collection of EILP diagnosis since 1986. [file 13047_2023_680_MOESM1_ESM.docx]

Additional file 1: Collection of EILP diagnosis since 1986

|  | Medial tibia stress syndrome |
| --- | --- |
|  | Chronic compartment syndrome |
|  | Stress fracture of the tibia |
|  | Superficial peroneal nerve entrapment syndrome |
|  | Sural nerve entrapment syndrome |
|  | Radiculopathy |
|  | Spinal stenosis |
|  | Popliteal artery entrapment syndrome |
|  | Popliteal artery stenosis |
|  | Muscle hernia |
|  | AV fistula |
|  | Osteoid osteoma |
|  | Osteoclastoma |
|  | Diabetic neuropathy and ischemia |
|  | McArdle syndrome |
|  | Eosinophilic anaemia |
|  | Sickle cell anaemia |
|  | Popliteal cyst rupture |
|  | Sarcoma of the soleus |
|  | Tibialis anterior muscle syndrome |
|  | Accessory soleus muscle syndrome |
|  | Accessory FHL muscle syndrome |
|  | Muscle tear |
|  | Myofascial tear |
|  | Infection |
|  | Delayed onset muscle soreness |
|  | CECS following acute compartment syndrome |
|  | Myositis ossificans |
|  | Hematoma |
|  | Neuromyotonia |
|  | Pseudoarthrosis and nerve impingement following L4 spinous process fracture |
|  | Garres sclerosing osteomyelitis |
|  | Necrobiosis lipodica dibeticorum |
|  | Cystic adventitial disease of the popliteal artery |
|  | Vitamin D deficiency |
|  | PAES due to entrapment by popliteus – Type 4 |
|  | Radicular leg pain due to secondaries in lumbar-sacral spine |
|  | Synostosis of proximal tibio-fibular joint |
|  | Klippel-Trenaunay syndrome |
|  | Stress fracture of the fibula |
