# Supplementary material for: Developing a diagnostic framework for patients presenting with Exercise Induced Leg Pain (EILP): a scoping review
Source: J Foot Ankle Res. 2023 Nov 21;16:82. doi: 10.1186/s13047-023-00680-6 (PMC10662794; doi:10.1186/s13047-023-00680-6)
Supplement: Supplementary file 3 — Additional file 3. Full search strategy. The full search strategy used within each database. [file 13047_2023_680_MOESM3_ESM.docx]

Additional file 3: Full search strategy

| **Search terms** | | |
| --- | --- | --- |
| **Construct** | | **Keywords** |
| Participants (conditions) | AND | “shin splints” OR EILP OR “exercise induced leg pain” OR “compartment syndrome” OR “chronic exertional compartment syndrome” OR “exertional compartment syndrome” OR CECS OR “anterior compartment syndrome” OR “posterior compartment syndrome” OR “lateral compartment syndrome” OR “stress fracture” OR “tibial stress fracture” OR “fibular stress fracture” OR “stress syndrome” OR “Medial tibial stress syndrome” OR “tibial stress syndrome” OR “tibial stress injury” OR MTSS OR “stress injury” OR “radicular leg” OR “leg radiculopathy” OR “lumbar radiculopathy” OR “lumbar radicular” OR “nerve entrapment syndrome” OR “neuropathy” OR “peroneal neuropathy” OR “superficial peroneal neuropathy” OR “arterial entrapment syndrome” OR “popliteal artery entrapment syndrome” OR (exercise pain) OR “Myopathy” OR “Glycogen Storage Disease Type V” OR “McArdle disease” OR “McArdle syndrome" |
| Participants (exercise) | AND | Exercise* OR active* OR sport* OR athlete* OR train* OR exertion OR practice* OR physical |
| Participants (anatomical location) | AND | Leg OR compartment* OR calf OR shin* OR “lower limb” OR tibia* OR fibula* OR fascia |
| outcomes | AND | Diagnoses* OR “differential diagnosis” OR presentation OR “Clinical presentation” OR detection OR “clinical reasoning” OR findings |
| Variables | NOT | In title: acute OR disease OR diabetes* OR foot OR feet OR thigh OR shoulder OR arm OR hand OR wrist OR back OR Raynaud’s OR cervical radiculopathy |

**Pubmed = 2511/ 2075 (English and human)**

**Entered:**

| All fields | AND | “shin splints” OR EILP OR “exercise induced leg pain” OR "Compartment Syndromes"[Mesh] OR “exertional compartment syndrome” OR CECS OR “posterior compartment syndrome” OR “lateral compartment syndrome” OR "Fractures, Stress"[Mesh] OR “tibial stress fracture” OR “fibular stress fracture” OR "Medial Tibial Stress Syndrome"[Mesh] OR “radicular leg” OR “leg radiculopathy” OR "Radiculopathy"[Mesh] OR “lumbar radiculopathy” OR “Nerve entrapment syndrome” OR “neuropathy” OR "Peroneal Neuropathies"[Mesh] OR “superficial peroneal neuropathy” OR “arterial entrapment syndrome” OR "Popliteal Artery Entrapment Syndrome"[Mesh] OR (exercise pain) |
| --- | --- | --- |
| Title/abs | AND | Exercise OR active OR sport OR athlete OR training OR exertion OR practising OR physical |
| Title/abs | AND | Leg OR compartment OR calf OR shin OR “lower limb” OR tibia OR fibula OR fascia |
| All fields | AND | Diagnos* OR “differential diagnosis” OR presentation OR “Clinical presentation” OR detection OR “clinical reasoning” OR findings |
| Title | NOT | acute OR disease OR diabetes OR foot OR feet OR thigh OR shoulder OR arm OR hand OR wrist OR back OR Raynaud’s OR cervical radiculopathy |
| All fields | NOT | Acetylcholinesterase OR oxidative metabolic OR education |

**Interepted:**

**((((("shin splints" OR EILP OR "exercise induced leg pain" OR "Compartment Syndromes"[Mesh] OR "exertional compartment syndrome" OR CECS OR "posterior compartment syndrome" OR "lateral compartment syndrome" OR "Fractures, Stress"[Mesh] OR "tibial stress fracture" OR "fibular stress fracture" OR "Medial Tibial Stress Syndrome"[Mesh] OR "radicular leg" OR "leg radiculopathy" OR "Radiculopathy"[Mesh] OR "lumbar radiculopathy" OR "Nerve entrapment syndrome" OR "neuropathy" OR "Peroneal Neuropathies"[Mesh] OR "superficial peroneal neuropathy" OR "arterial entrapment syndrome" OR "Popliteal Artery Entrapment Syndrome"[Mesh] OR (exercise pain)) AND (Exercise[Title/Abstract] OR active[Title/Abstract] OR sport[Title/Abstract] OR athlete[Title/Abstract] OR training[Title/Abstract] OR exertion[Title/Abstract] OR practising[Title/Abstract] OR physical[Title/Abstract])) AND (Leg[Title/Abstract] OR compartment[Title/Abstract] OR calf[Title/Abstract] OR shin[Title/Abstract] OR "lower limb"[Title/Abstract] OR tibia[Title/Abstract] OR fibula[Title/Abstract] OR fascia[Title/Abstract])) AND (Diagnos* OR "differential diagnosis" OR presentation OR "Clinical presentation" OR detection OR "clinical reasoning" OR findings)) NOT (acute[Title] OR disease[Title] OR diabetes[Title] OR foot[Title] OR feet[Title] OR thigh[Title] OR shoulder[Title] OR arm[Title] OR hand[Title] OR wrist[Title] OR back[Title] OR Raynaud’s[Title] OR cervical radiculopathy[Title])) NOT (Acetylcholinesterase OR oxidative metabolic OR education)**

**((((("shin splints" OR EILP OR "exercise induced leg pain" OR "Compartment Syndromes"[Mesh] OR "Chronic Exertional Compartment Syndrome"[Mesh] OR "exertional compartment syndrome" OR CECS OR "Anterior Compartment Syndrome"[Mesh] OR "posterior compartment syndrome" OR "lateral compartment syndrome" OR "Fractures, Stress"[Mesh] OR "tibial stress fracture" OR "fibular stress fracture" OR "Medial Tibial Stress Syndrome"[Mesh] OR "radicular leg" OR "leg radiculopathy" OR "Radiculopathy"[Mesh] OR "lumbar radiculopathy" OR "Nerve entrapment syndrome" OR "neuropathy" OR "Peroneal Neuropathies"[Mesh] OR "superficial peroneal neuropathy" OR "arterial entrapment syndrome" OR "Popliteal Artery Entrapment Syndrome"[Mesh] OR (exercise pain)) AND (Exercise[Title/Abstract] OR active[Title/Abstract] OR sport[Title/Abstract] OR athlete[Title/Abstract] OR training[Title/Abstract] OR exertion[Title/Abstract] OR practising[Title/Abstract] OR physical[Title/Abstract])) AND (Leg[Title/Abstract] OR compartment[Title/Abstract] OR calf[Title/Abstract] OR shin[Title/Abstract] OR "lower limb"[Title/Abstract] OR tibia[Title/Abstract] OR fibula[Title/Abstract] OR fascia[Title/Abstract])) AND (Diagnos* OR "differential diagnosis" OR presentation OR "Clinical presentation" OR detection OR "clinical reasoning" OR findings)) NOT (Acetylcholinesterase OR oxidative metabolic OR education)) NOT (acute[Title] OR disease[Title] OR diabetes[Title] OR foot[Title] OR feet[Title] OR thigh[Title] OR shoulder[Title] OR arm[Title] OR hand[Title] OR wrist[Title] OR back[Title] OR Raynaud’s[Title] OR cervical radiculopathy[Title]) Filters: English, Humans**

**Embase = 1137/ 983(English/human)**

**Entered:**

| All fields | AND | “shin splints” OR EILP OR “exercise induced leg pain” OR “compartment syndrome” OR “chronic exertional compartment syndrome” OR “exertional compartment syndrome” OR CECS OR “anterior compartment syndrome” OR “posterior compartment syndrome” OR “lateral compartment syndrome” OR “stress fracture” OR “tibial stress fracture” OR “fibular stress fracture” OR “stress syndrome” OR “Medial tibial stress syndrome” OR “tibial stress syndrome” OR “tibial stress injury” OR MTSS OR “stress injury” OR “radicular leg” OR “leg radiculopathy” OR “lumbar radiculopathy” OR “lumbar radicular” OR “nerve entrapment syndrome” OR “neuropathy” OR “peroneal neuropathy” OR “superficial peroneal neuropathy” OR “arterial entrapment syndrome” OR “popliteal artery entrapment syndrome” OR (exercise pain) |
| --- | --- | --- |
| Title/abs/key | AND | Exercis* OR active* OR sport* OR athlete* OR train* OR exertion OR practis* OR physical |
| Title/abs/key | AND | Leg OR compartment* OR calf OR shin* OR “lower limb” OR tibia* OR fibula* OR fascia |
| All fields | AND | Diagnos* OR “differential diagnosis” OR presentation OR “Clinical presentation” OR detection OR “clinical reasoning” OR findings |
| All Fields | NOT | acute OR disease OR diabet* OR foot OR feet OR thigh OR shoulder OR arm OR hand OR wrist OR back OR Raynauds |
| title | NOT | Acetylcholinesterase OR oxidative metabolic OR education |

**Interepted:**

('shin splints' OR eilp OR 'exercise induced leg pain' OR 'compartment syndrome' OR 'chronic exertional compartment syndrome' OR 'exertional compartment syndrome' OR cecs OR 'anterior compartment syndrome' OR 'posterior compartment syndrome' OR 'lateral compartment syndrome' OR 'stress fracture' OR 'tibial stress fracture' OR 'fibular stress fracture' OR 'stress syndrome' OR 'medial tibial stress syndrome' OR 'tibial stress syndrome' OR 'tibial stress injury' OR mtss OR 'stress injury' OR 'radicular leg' OR 'leg radiculopathy' OR 'lumbar radiculopathy' OR 'lumbar radicular' OR 'nerve entrapment syndrome' OR 'neuropathy' OR 'peroneal neuropathy' OR 'superficial peroneal neuropathy' OR 'arterial entrapment syndrome' OR 'popliteal artery entrapment syndrome' OR (exercise AND pain)) AND (exercis*:ti,ab,kw OR active*:ti,ab,kw OR sport*:ti,ab,kw OR athlete*:ti,ab,kw OR train*:ti,ab,kw OR exertion:ti,ab,kw OR practis*:ti,ab,kw OR physical:ti,ab,kw) AND (leg:ti,ab,kw OR compartment*:ti,ab,kw OR calf:ti,ab,kw OR shin*:ti,ab,kw OR 'lower limb':ti,ab,kw OR tibia*:ti,ab,kw OR fibula*:ti,ab,kw OR fascia:ti,ab,kw) AND (diagnos* OR 'differential diagnosis' OR presentation OR 'clinical presentation' OR detection OR 'clinical reasoning' OR findings) NOT (acute OR disease OR diabet* OR foot OR feet OR thigh OR shoulder OR arm OR hand OR wrist OR back OR raynauds) NOT (acetylcholinesterase:ti OR 'oxidative metabolic':ti OR education:ti)

('shin splints' OR eilp OR 'exercise induced leg pain' OR 'compartment syndrome' OR 'chronic exertional compartment syndrome' OR 'exertional compartment syndrome' OR cecs OR 'anterior compartment syndrome' OR 'posterior compartment syndrome' OR 'lateral compartment syndrome' OR 'stress fracture' OR 'tibial stress fracture' OR 'fibular stress fracture' OR 'stress syndrome' OR 'medial tibial stress syndrome' OR 'tibial stress syndrome' OR 'tibial stress injury' OR mtss OR 'stress injury' OR 'radicular leg' OR 'leg radiculopathy' OR 'lumbar radiculopathy' OR 'lumbar radicular' OR 'nerve entrapment syndrome' OR 'neuropathy' OR 'peroneal neuropathy' OR 'superficial peroneal neuropathy' OR 'arterial entrapment syndrome' OR 'popliteal artery entrapment syndrome' OR (exercise AND pain)) AND (exercis*:ti,ab,kw OR active*:ti,ab,kw OR sport*:ti,ab,kw OR athlete*:ti,ab,kw OR train*:ti,ab,kw OR exertion:ti,ab,kw OR practis*:ti,ab,kw OR physical:ti,ab,kw) AND (leg:ti,ab,kw OR compartment*:ti,ab,kw OR calf:ti,ab,kw OR shin*:ti,ab,kw OR 'lower limb':ti,ab,kw OR tibia*:ti,ab,kw OR fibula*:ti,ab,kw OR fascia:ti,ab,kw) AND (diagnos* OR 'differential diagnosis' OR presentation OR 'clinical presentation' OR detection OR 'clinical reasoning' OR findings) NOT (acute OR disease OR diabet* OR foot OR feet OR thigh OR shoulder OR arm OR hand OR wrist OR back OR raynauds) NOT (acetylcholinesterase:ti OR 'oxidative metabolic':ti OR education:ti) AND [english]/lim AND [humans]/lim

**SCOPUS = 4310/ 3116(human/English)**

**Entered:**

| All fields | AND | “shin splints” OR EILP OR “exercise induced leg pain” OR “compartment syndrome” OR “chronic exertional compartment syndrome” OR “exertional compartment syndrome” OR CECS OR “anterior compartment syndrome” OR “posterior compartment syndrome” OR “lateral compartment syndrome” OR “stress fracture” OR “tibial stress fracture” OR “fibular stress fracture” OR “stress syndrome” OR “Medial tibial stress syndrome” OR “tibial stress syndrome” OR “tibial stress injury” OR MTSS OR “stress injury” OR “radicular leg” OR “leg radiculopathy” OR “lumbar radiculopathy” OR “lumbar radicular” OR “nerve entrapment syndrome” OR “neuropathy” OR “peroneal neuropathy” OR “superficial peroneal neuropathy” OR “arterial entrapment syndrome” OR “popliteal artery entrapment syndrome” OR (exercise pain) |
| --- | --- | --- |
| Title/abs | AND | Exercis* OR active* OR sport* OR athlete* OR train* OR exertion OR practis* OR physical |
| Title/abs | AND | Leg OR compartment* OR calf OR shin* OR “lower limb” OR tibia* OR fibula* OR fascia |
| All fields | AND | Diagnos* OR “differential diagnosis” OR presentation OR “Clinical presentation” OR detection OR “clinical reasoning” OR findings |
| Title/abs | NOT | acute OR disease OR diabet* OR foot OR feet OR thigh OR shoulder OR arm OR hand OR wrist OR back OR Raynauds |
| All fields | NOT | Acetylcholinesterase OR oxidative metabolic OR education |

**Interepted:**

ALL ( "shin splints"  OR  eilp  OR  "exercise induced leg pain"  OR  "compartment syndrome"  OR  "chronic exertional compartment syndrome"  OR  "exertional compartment syndrome"  OR  cecs  OR  "anterior compartment syndrome"  OR  "posterior compartment syndrome"  OR  "lateral compartment syndrome"  OR  "stress fracture"  OR  "tibial stress fracture"  OR  "fibular stress fracture"  OR  "stress syndrome"  OR  "Medial tibial stress syndrome"  OR  "tibial stress syndrome"  OR  "tibial stress injury"  OR  mtss  OR  "stress injury"  OR  "radicular leg"  OR  "leg radiculopathy"  OR  "lumbar radiculopathy"  OR  "lumbar radicular"  OR  "nerve entrapment syndrome"  OR  "neuropathy"  OR  "peroneal neuropathy"  OR  "superficial peroneal neuropathy"  OR  "arterial entrapment syndrome"  OR  "popliteal artery entrapment syndrome"  OR  ( exercise  AND pain ) )  AND  TITLE-ABS ( exercis*  OR  active*  OR  sport*  OR  athlete*  OR  train*  OR  exertion  OR  practis*  OR  physical )  AND  TITLE-ABS ( leg  OR  compartment*  OR  calf  OR  shin*  OR  "lower limb"  OR  tibia*  OR  fibula*  OR  fascia )  AND  ALL ( diagnos*  OR  "differential diagnosis"  OR  presentation  OR  "Clinical presentation"  OR  detection  OR  "clinical reasoning"  OR  findings )  AND NOT  ALL ( acetylcholinesterase  OR  oxidative  AND metabolic  OR  education )  AND NOT  TITLE-ABS ( acute  OR  disease  OR  diabet*  OR  foot  OR  feet  OR  thigh  OR  shoulder  OR  arm  OR  hand  OR  wrist  OR  back  OR  raynauds )
